# The multifaceted functions of cGAS

**DOI:** 10.1093/jmcb/mjac031

**Published:** 2022-05-10

**Authors:** Haipeng Liu, Fei Wang, Yajuan Cao, Yifang Dang, Baoxue Ge

**Affiliations:** Clinical and Translational Research Center, Shanghai Pulmonary Hospital, School of Medicine, Tongji University, Shanghai 200433, China; Shanghai Key Laboratory of Tuberculosis, Shanghai Pulmonary Hospital, School of Medicine, Tongji University, Shanghai 200433, China; Central Laboratory, Shanghai Pulmonary Hospital, School of Medicine, Tongji University, Shanghai 200433, China; Clinical and Translational Research Center, Shanghai Pulmonary Hospital, School of Medicine, Tongji University, Shanghai 200433, China; Shanghai Key Laboratory of Tuberculosis, Shanghai Pulmonary Hospital, School of Medicine, Tongji University, Shanghai 200433, China; Clinical and Translational Research Center, Shanghai Pulmonary Hospital, School of Medicine, Tongji University, Shanghai 200433, China; Clinical and Translational Research Center, Shanghai Pulmonary Hospital, School of Medicine, Tongji University, Shanghai 200433, China; Clinical and Translational Research Center, Shanghai Pulmonary Hospital, School of Medicine, Tongji University, Shanghai 200433, China; Shanghai Key Laboratory of Tuberculosis, Shanghai Pulmonary Hospital, School of Medicine, Tongji University, Shanghai 200433, China

**Keywords:** cyclic GMP–AMP synthase (cGAS), stimulator of interferon genes (STING), DNA sensing, innate immunity, micronucleophagy

## Abstract

Pattern recognition receptors are critical for the sensing of pathogen-associated molecular patterns or danger-associated molecular patterns and subsequent mounting of innate immunity and shaping of adaptive immunity. The identification of 2′3′-cyclic guanosine monophosphate–adenosine monophosphate (cGAMP) synthase (cGAS) as a major cytosolic DNA receptor is a milestone in the field of DNA sensing. The engagement of cGAS by double-stranded DNA from different origins, including invading pathogens, damaged mitochondria, ruptured micronuclei, and genomic DNA results in the generation of cGAMP and activation of stimulator of interferon genes, which thereby activates innate immunity mainly characterized by the activation of type I interferon response. In recent years, great progress has been made in understanding the subcellular localization and novel functions of cGAS. In this review, we particularly focus on summarizing the multifaceted roles of cGAS in regulating senescence, autophagy, cell stemness, apoptosis, angiogenesis, cell proliferation, antitumor effect, DNA replication, DNA damage repair, micronucleophagy, as well as cell metabolism.

## Introduction

Innate immunity provides an important first line of defense against invading pathogens or harmful damages, which relies on pattern recognition receptors (PRRs) expressed on the surface or inside the immune cells to recognize pathogen-associated molecular patterns (PAMPs) or danger-associated molecular patterns (DAMPs) (Briard et al., 2020). In 2008, the stimulator of interferon genes (STING) was identified as a new key protein located in the endoplasmic reticulum (ER) to initiate an innate immune response (Zhong et al., 2008; Sun et al., 2009), which can be activated by DNA to exert immunomodulatory effects (Ishikawa and Barber, 2008; Abe and Barber, 2014). It was found that STING does not bind directly to DNA but binds to intracellular second messengers, such as cyclic guanosine diphosphate and cyclic adenosine diphosphate produced by bacteria, which result in conformational changes of STING and recruit downstream signaling molecules to activate innate immune responses (Cong et al., 2019). However, in mammals, the activation mode of STING was not revealed until 2012. The team of Dr Zhijian James Chen identified 2′3′-cyclic guanosine monophosphate–adenosine monophosphate (cGAMP) as an endogenous second messenger capable of binding to STING and initiating a downstream type I interferon (IFN) response (Wu and Chen, 2014). Meanwhile, the team identified the enzyme catalyzing cGAMP synthesis from mouse fibroblast cell line L929 by biochemical isolation and quantitative mass spectrometry and named it cyclic GMP–AMP synthase (cGAS) (Sun et al., 2013). The discovery of cGAS was a milestone in the history of innate immunity research and opened a new door for the study of regulatory mechanisms of innate immune activation, especially DNA-mediated immune regulation.

## cGAS is a critical cytosolic DNA sensor

As a classical cytosolic DNA receptor, the engagement of cGAS by DNA activates its enzymatic activity to synthesize cGAMP using guanosine triphosphate (GTP) and adenosine triphosphate (ATP). cGAMP interacts with STING resident on the ER and results in the translocation of STING from the ER to the ER–Golgi intermediate compartment (ERGIC) or the Golgi apparatus. Then STING recruits IκB kinase (IKK) and TANK binding kinase 1 (TBK1), which activate important transcription factors including nuclear factor kappa-B (NF-κB) and interferon regulatory factor 3 (IRF3). Activated NF-κB and IRF3 enter the nucleus from the cytoplasm and initiate the expression of multiple cytokines including interferon-β (IFN-β) (Abe and Barber, 2014). cGAS thereby plays a key role in a variety of diseases including infection, autoimmune diseases, and cancer by modulating the immune response.

### Source of DNA recognized by cGAS

**Figure 1:**
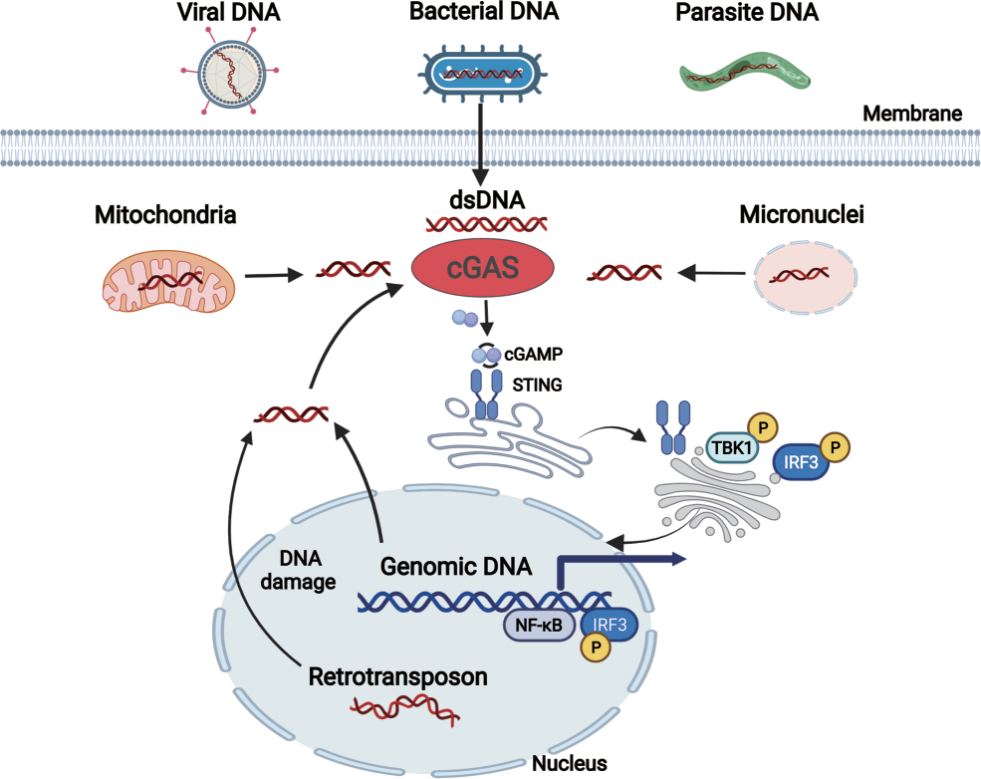
Diversified sources of DNA for the activation of cGAS. In addition to dsDNA from invading pathogens including viruses, bacteria, and parasites, DNA released from damaged mitochondria, ruptured micronuclei, genomic DNA, and retrotransposons are also engaged by cGAS to activate STING-mediated innate immune response.

Under non-healthy conditions, exogenous DNA from pathogens such as bacteria, viruses, and parasites is an important PAMP for activating cGAS. DNA of Nipah virus, measles virus, and herpes simplex virus type I (HSV-1) can be sensed by cGAS and activates STING-mediated type I IFN response (Iampietro et al., 2021; Reinert et al., 2021). Meanwhile, cGAS is critical for the induction of type I IFN response to infection by various bacteria such as *Chlamydia trachomatis* (Zhang et al., 2014), *Mycobacterium tuberculosis* (Watson et al., 2015), *Listeria monocytogenes* (Hansen et al., 2014), and *Neisseria gonorrhoeae* (Andrade et al., 2016). In addition to viral and bacterial DNA, cGAS is important for type I IFN production in response to parasite infection, including protozoans and worms (Hahn et al., 2018; Chen et al., 2022; Liang et al., 2022). Meanwhile, activation of the cGAS signaling pathway is not limited to mount a defense against invading pathogens; cGAS can be activated by its own DNA, including mitochondrial DNA (mtDMA), genomic DNA, and DNA in the micronuclei and retrotransposon (Li and Chen, 2018; Ablasser and Chen, 2019; Hopfner and Hornung, 2020). The activation of the cGAS–STING signaling pathway is a double-edged sword. When pathogenic microorganisms invade, the activation of this pathway helps host cells to rapidly produce a high level of IFN to resist the infection of pathogenic microorganisms. When a variety of genetic or environmental factors cause abnormal accumulation of self-DNA and mtDNA in the cytoplasm, it will lead to serious neurodegenerative diseases and autoimmune diseases, including systemic lupus erythematosus, rheumatoid arthritis, etc. (Ahn et al., 2012; Gall et al., 2012; Gao et al., 2015; Gray et al., 2015; An et al., 2017; Skopelja-Gardner et al., 2020). When a cell is subjected to stress or environmental damage, DNA confined to the nucleus or mitochondria can enter the cytoplasm and activate cGAS to trigger an immune response. Obesity leads to the release of mtDNA from adipose tissue cells to the cytoplasm, which leads to inflammatory responses through the activation of the cGAS–STING signaling pathway, and then induces insulin resistance and diabetes (Bai et al., 2017). In the septicemia mouse model, lipopolysaccharide (LPS) activates Gasdermin D and mediates the release of mtDNA into the cytoplasm in endothelial cells, which then activates the cGAS–STING signaling pathway, inducing inflammatory response, downregulated YAP1 signaling pathway, and the final failure in the regenerating ability of endothelial cells (Huang et al., 2020). Meanwhile, studies have shown that the mtDNA-dependent cGAS–STING signaling pathway is controlled by metabolism and induced by cytosine deficiency (Sprenger et al., 2021). Studies have shown that endogenous retrotransposons are widely activated during aging (De Cecco et al., 2013; Simon et al., 2019), and recognition of retrotransposons by cGAS may lead to aging-related inflammation and pathology (Figure 1; De Cecco et al., 2013; Simon et al., 2019).

At the structural level, cGAS is more capable of recognizing double-stranded DNA (dsDNA). Also, the binding strength of cGAS to dsDNA depends mainly on the length of dsDNA, independent of its nucleotide sequence and composition. Relative to dsDNA, cGAS has a weak recognition capacity for single-stranded DNA (ssDNA) and does not recognize RNA in general (Tao et al., 2016). However, cGAS was found to be activated by ssDNA from type I human immunodeficiency virus (HIV) in a concentration-dependent manner, particularly Y-type DNA (Herzner et al., 2015). In addition, the RNA:DNA hybrid strands present in the life cycle of a large number of DNA viruses can also significantly activate cGAS (Mankan et al., 2014). Current studies suggest that dsDNA, ssDNA, and RNA:DNA hybrid strands can be recognized by cGAS under certain conditions, thus activating the innate immune response.

### Mode and structural basis of cGAS activation

Human cGAS is a protein consisting of 522 amino acids, and its N-terminal structural domain consists of amino acids 1–160 and may contain three distinct DNA-binding sites (Li et al., 2013). cGAS has a C-terminus consisting of amino acids 161–522, which includes not only an NTase core structural domain and a highly conserved male abnormal 21 (Mab21) domain that was first identified in *Caenorhabditis elegans* (Sun et al., 2013), but also a site-C dsDNA-binding domain (Cao et al., 2020). The mutation of the Mab21 structural domain of cGAS resulted in its incapable of inducing IFN-β expression, suggesting that it plays an important role in cGAS function (Sun et al., 2013). The Mab21 structural domain consists of two parts: the first part consists of two α-helices surrounding two β-folds, while the second part contains four α-helices. These two parts are connected by a spine-like α-helix, two connecting helices, and an active site. On the opposite side of the active site, there is a concave platform with a zinc finger structure at one end. During DNA recognition, cGAS forms a dimer that binds to the phosphoglycan backbone of DNA. The DNA duplex is attached to the spine-like α-helix of cGAS and the zinc finger structure, and the cGAS dimer forms a ladder network in a head-to-head pattern next to the DNA, eventually forming a 2:2 complex (Civril et al., 2013). The binding of DNA to cGAS causes significant conformational changes; the catalytic site shifts and opens the catalytic pocket, and ATP and GTP enter the pocket. cGAS exerts enzymatic activity to catalyze the synthesis of cGAMP from ATP and GTP. The synthesized cGAMP enters the cytoplasm and binds to STING on the ER membrane. STING preforms a V-shaped capsule composed of dimers before binding to cGAMP, and the C-terminal domain of two STINGs is exposed in the cytoplasm. When cGAMP binds, the conformation of STING changes and the V-shaped dimer becomes more closed and forms a lid over cGAMP, keeping the binding of both stable. Upon binding to cGAMP, STING translocates from the ER to the ERGIC, recruiting downstream TBK1 and IKK and initiating the expression of cytokines including type I IFN (Ablasser and Chen, 2019). Another site-C dsDNA-binding domain is mainly composed of three marker fragments, namely α-region (261–286), KRKR-loop (299–302), and KKH-loop (427–432). This DNA-binding region promotes multivalency-induced liquid-phase condensation and the generation of cGAMP. Notably, the discovery of this binding domain provides a powerful platform for the structural insights into nucleosome inhibition of cGAS activity, thus providing opportunities for the treatment of autoimmune diseases (Cao et al., 2020).

Phase separation is an important aggregated partitioning mechanism of intracellular biochemical reactions. cGAS-mediated innate immune activation is also regulated by phase separation. cGAS has a positively charged and disordered N terminus, which can facilitate its binding to negatively charged DNA, leading to liquid–liquid phase separation of the cGAS–DNA complex. This phase separation of the cGAS–DNA complex creates a relatively independent environment that can avoid the inhibition of cGAS by negative regulators such as nucleic acid exonucleases three prime repair exonuclease 1 (TREX1) and BAF, facilitating the production of cGAMP and the transduction of innate immune signals. Long sequences of DNA are more able than short sequences of DNA to promote the liquid–liquid phase separation of the cGAS–DNA complex, thus enhancing the enzymatic activity of cGAS, which explains why the degree of cGAS activation is related to the sequence length of DNA (Zhou et al., 2018). Importantly, recent work demonstrated that liquid–liquid phase separation of the cGAS–DNA complex can be interrupted by ORF52 and VP22 proteins of α-herpesvirus and γ-herpesvirus, thereby inhibiting innate immune activation to achieve immune escape (Xu et al., 2021).

Previous studies have shown that cGAS recognizes and binds to DNA in the presence of Mg^2+^ and catalyzes the formation of cGAMP to activate innate immunity (Civril et al., 2013). However, it has recently been found that Mn^2+^ can directly activate cGAS enzymatic activity to synthesize cGAMP. Either mutation of lysine 394 (an amino acid critical for cGAS dimerization), mutation of lysine 384 (an amino acid essential for cGAS–DNA binding), or mutation of both completely inhibit the activation of DNA on cGAS, but these mutations do not affect the Mn^2+^-induced cGAS activation, indicating that the activation of Mn^2+^ has nothing to do with the traditional dsDNA-induced cGAS dimerization (Zhao et al., 2020). The mechanism study revealed that Mn^2+^ forms cGAS–Mn^2+^–pppGpG ternary complex with cGAS and GTP. A unique α-helix is formed at the active site of cGAS during the activation process, which broadens its catalytic site. Then, cGAS undergoes a conformational change very similar to traditional DNA activation, activating its enzymatic activity to synthesize cGAMP (Lv et al., 2020; Zhao et al., 2020).

### Cellular localization of cGAS

cGAS plays an important regulatory role in innate immunity as an important PRR. However, its overt activation also causes diseases including inflammation and autoimmune diseases (Barnett et al., 2019). Therefore, the tight regulation of cGAS is particularly important for its proper function. In addition to the original identification of cGAS in the cytosol, cGAS was found to localize to the micronuclei in the cytoplasm, and when the micronuclear membrane is ruptured, cGAS recognizes DNA within the micronuclei to activate innate immunity (Mackenzie et al., 2017). Meanwhile, DNA damage caused by cancer treatment leads to an increase in the number of micronuclei in the cytoplasm, which in turn activates cGAS and recruits immune cells to attack cancer cells (Harding et al., 2017). We have also shown that cGAS regulates the autophagic process of micronuclei as a micronucleophagy receptor (Zhao et al., 2021). It was shown that cGAS is prone to accumulate on the micronuclei harboring DNA damage (Mankan et al., 2014; Harding et al., 2017; Zhao et al., 2021). It was hypothesized that the micronuclei localization of cGAS might be related to DNA damage in micronuclei. A growing body of data shows that during mitosis, the nuclear envelope ruptures and cGAS dissociates from the chromosome, whereas micronuclei tend to have a fragile nuclear envelope, resulting in the exposure of their dsDNA to cGAS (Yang et al., 2017). cGAS enters micronuclei, where cGAS mediates downstream immune responses in a timely and cell cycle-dependent manner (Gao et al., 2020). cGAS in micronuclei can also oligomerize to play a role in immune defense (Li et al., 2021b). In addition, very recent work revealed the cell membrane-localizing characteristics of cGAS: in human and mouse macrophages, cGAS is localized at the cell membrane in the resting state (Barnett et al., 2019). Mechanistic studies revealed that the N-terminal of cGAS is highly polarized and positively charged in the cytoplasm, while phosphatidylinositide phosphates (PIPs) on the cell membrane are negatively charged (Balla, 2013). The positively charged N-terminal structural domain of cGAS and the negatively charged PIPs interact through electrostatic effects, thus retaining cGAS on the cell membrane. This localization avoids the activation of cGAS by its own trace DNA, which may lead to autoimmune diseases (Barnett et al., 2019).

**Figure 2:**
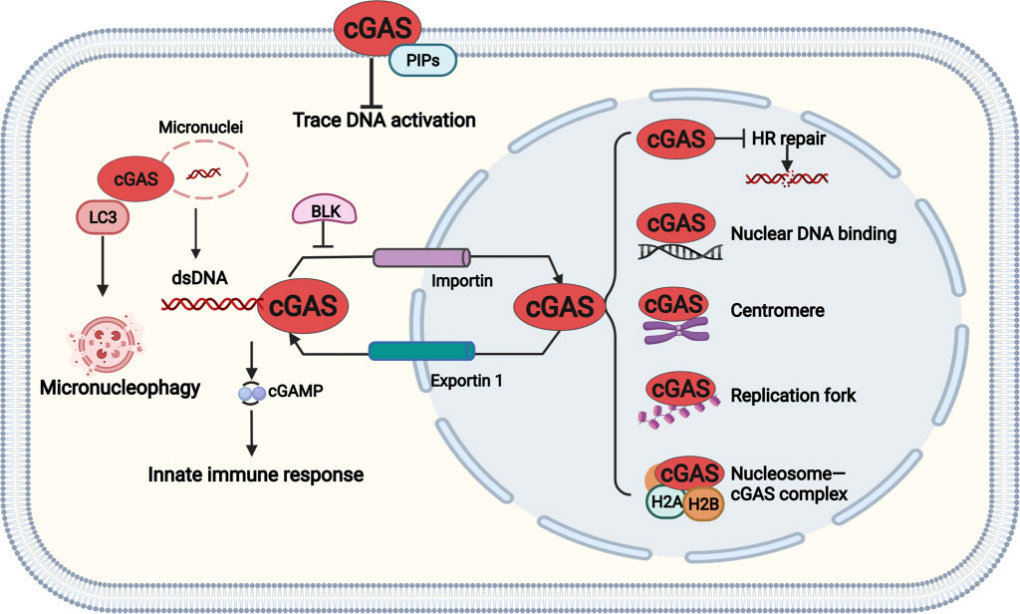
Regulation of cellular localization of cGAS. cGAS was originally found to be activated by dsDNA in the cytosol, which leads to the generation of the second messenger 2′3′-cGAMP and the induction of innate immune response. Now, it is generally accepted that cGAS is also located in the nucleus. Nuclear cGAS has been demonstrated to be critical in regulating DNA damage repair and replication fork stability. Nuclear cGAS is inactivated by association with nucleosome H2A and H2B. The property of chromosome binding and centromere location of cGAS has also been reported, though the functions remain elusive. In addition, the shuttle of cGAS between the cytosol and the nucleus is tightly regulated. NES is present in cGAS and mediates its translocation from the nucleus to the cytosol, which is critical for the DNA sensor function of cytosolic cGAS. NLS is also present in cGAS and its interaction with Importin is critical for the nuclear translocation of cGAS under genotoxic stresses. Moreover, the protein tyrosine kinase BLK-mediated phosphorylation of cGAS on tyrosine 215 is critical for its cytosolic retention. The electrostatic effects between the negative charge of PIPs on the membrane and the positive charge of the N-terminal structural domain of cGAS are critical for the membrane localization of cGAS in immune cells, which avoids the activation of cGAS by its own trace DNA in resting state. cGAS has also been found to locate on micronuclei, which mediates the sensing of dsDNA from micronuclei upon rupture. In addition. cGAS induces autophagy and lysosomal degradation of micronuclei by directly interacting with ATG8/LC3 in a STING-independent manner, and thereby serves as a micronucleophagy receptor. Though the membrane rupture and DNA damage of micronuclei have been implicated to be important for the recruitment of cGAS to micronuclei, the key factors involved in regulating the recruitment process remain to be identified.

Recently, the localization of cGAS in both the cytoplasm and nucleus of HeLa and THP-1 cells was observed and a nuclear export signal (NES) at the amino acids 169–174 of cGAS was identified, which is critical for the cytosolic translocation of cGAS from the nuclei in a chromosome region maintenance 1 (CRM1)-dependent manner. If CRM1 is inhibited by the addition of lepromycin B or if the amino acid site in the NES is directly mutated so that the entry of cGAS into the cytoplasm is blocked, the recognition of DNA by cGAS will be inhibited and it will not be able to induce the production of IFN, indicating that the function of cGAS to recognize and bind to DNA in the cytoplasm is dependent on the cytoplasmic translocation controlled by the NES (Sun et al., 2021). However, one limitation of this study is that the amino acids 169–174 are required for DNA binding of cGAS, which may directly impair cGAS activation regardless of the regulation of its cellular distribution. A magnitude of studies demonstrated that cGAS has nuclear localization (Liu et al., 2018; Xia et al., 2018). Existing in the nucleus of the hematopoietic stem cells, cGAS can bind with circular RNA antagonist for cGAS and lose enzymatic activity, thus maintaining homeostasis of hematopoietic stem cells (Xia et al., 2018). Intranuclear cGAS of dendritic cells and macrophages can synergistically enhance the recognition of HIV together with nuclear protein NONO (Lahaye et al., 2018). Under the pressure of DNA damage (etoposide, camptothecin, and H_2_O_2_ treatment), cGAS can enter the nucleus rapidly and in large quantities, and its entry into the nucleus does not depend on the rupture of the nuclear membrane. Moreover, the DNA-recognizing ability and enzymatic activity of cGAS are not involved in its nuclear entry regulation. Under physiological conditions, B-lymphoid tyrosine kinase (BLK) interacts with cGAS in the cytoplasm, causing phosphorylation modification of the tyrosine at position 215 of cGAS, which in turn maintains the cytoplasmic localization of cGAS. When cells undergo DNA damage, tyrosine 215 of cGAS is dephosphorylated and, subsequently, cGAS translocates from the cytoplasm to the nucleus and is recruited to the DNA double-strand break (DSB) site to inhibit the homologous recombination (HR)-mediated DNA damage repair process (Liu et al., 2018). In addition, cGAS has a nuclear localization sequence (NLS) that regulates its translocation into the nucleus by interacting with Importin α. This study is the first to demonstrate that cGAS entry into the nucleus is a regulated process (Liu et al., 2018). Recent studies have shown that both cGAS entry into the nucleus and the DNA-binding ability are necessary to slow down cell proliferation. Meanwhile, loss of cGAS promotes replication fork progression, which in turn leads to fork instability. Although the high replicating state of cGAS-deficient cells can activate the ataxia telangiectasia mutated and Rad3-related protein–checkpoint kinase 1 pathway, it cannot hinder the rapid proliferation of cells, and the cells are in a state of genomic instability, which in turn leads to a significant increase in the sensitivity of cells to radiotherapy and chemotherapy (Chen et al., 2020). In summary, on one hand, the precise regulation of cGAS localization in the cell can both ensure the normal function of cGAS under pathological conditions and avoid autoimmune diseases caused by abnormal activation of cGAS under normal conditions; on the other hand, the precise localization and cellular compartalization of cGAS guarantee its proper functions (Figure 2).

## Diversified functions of cGAS

### cGAS regulates innate immunity

**Figure 3:**
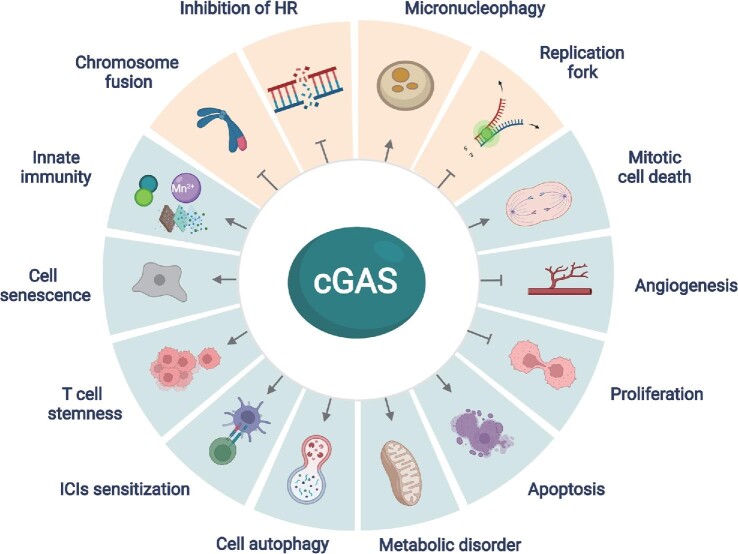
Diversified functions of cGAS. In addition to the canonical function as a cytosolic DNA sensor in the modulation of innate immunity, multifaceted roles of cGAS have been revealed. Importantly, cGAS is critical in regulating various biological processes including DNA damage repair, DNA replication, chromosome fusion, and micronucleophagy in a STING-independent manner (highlighted as light yellow background).

Early studies on cGAS mainly focused on its classical function in innate immune regulation. When cGAS recognizes pathogen DNA in the cytoplasm of cells, the cGAS–STING signaling pathway is activated and plays an important role in host defense against pathogen infection and tumor development. As mentioned above, cGAS is critical for the induction of type I IFN response and innate immunity in response to invading pathogens including viruses, bacteria, and parasites. Meanwhile, cGAS can recognize tumor cell DNA and produce large amounts of IFN-β, which in turn enhances antitumor immunity to control tumor development (Yum et al., 2020; Zheng et al., 2020). In human colon carcinoma (Xia et al., 2016), melanoma (Falahat et al., 2019), glioma (Ohkuri et al., 2014), and hepatoma cell lines (Thomsen et al., 2020), tumor cells can evolve strategies to inhibit the activation of the cGAS–STING signaling pathway and reduce the production of IFN-β, which is conducive to the survival of tumor cells. Therefore, the cGAS–STING signaling pathway is crucial to defend pathogen infection and tumor progression. Notably, cGAS not only recognizes the DNA of pathogens in the cytoplasm but also can be activated by the cell's own DNA, such as DNA released from mitochondria and the nucleus. When genomic instability leads to DNA damage or mitochondrial stress, the activation of cGAS can lead to autoimmune diseases and metabolic dysfunctions (Figure 3; Li and Chen, 2018; Bai and Liu, 2019).

### cGAS regulates cellular senescence

Cellular senescence is an irreversible process that has a crucial regulatory role in the normal life cycle of the organism. There are many factors that induce cellular senescence, intrinsic factors such as DNA damage and telomerase shortening (Liu et al., 2019b) and extrinsic factors including UV irradiation, hypoxia, toxic reagents, and acute stressful environments, such as highly carcinogenic substances (Zhang et al., 2018). Senescent cells secrete a variety of cytokines, such as inflammatory cytokines, growth factors, and proteases, a phenomenon known as senescence-associated secretory phenotype (SASP). It was found that DNA damage leads to the production of IFNs, which in turn induces cellular senescence. The deletion of the cGAS gene in mouse embryonic fibroblasts significantly slowed down cellular senescence and progressed toward immortalization. Subsequently, the investigators induced fibroblast senescence using etoposide, an anticancer drug that has the ability to strongly induce cellular senescence, and examined the effect of cGAS on cellular senescence during this process. It was found that cGAS is essential for the secretory phenotype of cellular senescence and cGAS deficiency significantly inhibits the expression and secretion of senescence-associated cytokines including IL-6, IL-8, IFN-β, and CXCL10 (Yang et al., 2017). cGAS activation, as well as STING dimer formation, was found in senescent cells, demonstrating that the cGAS–STING pathway is involved in the regulation of cellular senescence (Dou et al., 2017). In addition, cGAS also regulates cellular senescence in a STING-independent manner (Li et al., 2022). During mitosis, cGAS interacts with CDK1 and co-localizes to the ends of broken chromosomes. cGAS inhibits non-homologous end joining (NHEJ)-mediated DNA repair by regulating ring finger protein 8 recruitment, which in turn reduces the occurrence of end fusions on broken chromosomes and promotes replicative senescence. This role of cGAS in promoting replicative senescence is not dependent on the traditional cGAS–STING signaling pathway. The cGAS–STING signaling pathway-mediated SASP plays a double-edged role in tumorigenesis and progression. On one hand, cytokines secreted by senescent cells can activate immune cells within the tumor, thus promoting tumor immunity and inhibiting tumor development; on the other hand, in chronic inflammation, the continued secretion of related factors such as IL-1β and matrix metalloprotein by senescent cells promotes tumorigenesis (Loo et al., 2020). This dual regulatory role may be related to the specific tumor microenvironment. In addition, activation of the cGAS–STING pathway by cellular senescence may promote the induction of early age-related macular degeneration lesions (AMD), and thus this pathway may be a potential new target for the treatment of early AMD (Wu et al., 2019). Therefore, cGAS regulates the senescence of cells and modulates the development of multiple diseases through a STING-dependent or STING-independent manner (Figure 3).

### cGAS regulates cellular autophagy

Autophagy is a process of delivering intracellular substances to lysosomes for degradation and reuse, which contributes to the intracellular recycling of energy and substances (Mizushima and Komatsu, 2011). It was found that the cGAS–STING pathway functions to induce autophagy, which is independent of the production of IFNs and other inflammatory factors downstream of STING. Upon infection-mediated activation of cGAS, the synthesized cGAMP binds to STING, which interacts with SEC24C and buds out of the ER into coat protein complex II vesicles to form the ERGIC. ERGIC serves as a membrane source for WD repeat domain, phosphoinositide interacting 2 recruitment and microtubule-associated protein 1A/1B–light chain 3 (LC3) lipidation, which contributes to autophagic vesicle formation, thereby degrading viral RNA or DNA in the cytoplasm by autophagy and reducing the inflammatory response (Gui et al., 2019). STING in sea anemones, a species that existed 500 million years ago, structurally lacks the C-terminal TBK1 activation domain but is still able to promote autophagic vesicle formation after cGAMP stimulation, suggesting that the induction of autophagy is an ancient and highly conserved function of the cGAS–STING pathway, predating the vertebrate function of regulating type I IFN expression (Gui et al., 2019; Figure 3).

### cGAS regulates cell stemness

Stem cells are a class of cell populations with an unlimited capacity for self-renewal and differentiation (Wen and Tang, 2016). T cells also have stem cell-like properties, and these T cells are self-renewing, infinitely proliferative, and pluripotent (Vodnala et al., 2019) and play an important role in immune checkpoint blockade-mediated tumor immunotherapy. T cell factor 1 (TCF1) is a transcriptional factor encoded by *Tcf7* that regulates embryonic development and tissue stem cell self-renewal as a direct downstream effector of the WNT/β-catenin pathway (Kim et al., 2020). TCF1 therefore serves as a hallmark of stem T cells. In tumor immunotherapy, TCF1^+^ CD8 T cells are a subset of T cells that positively correlate with the efficacy of PD-1 immune checkpoint inhibitor (ICI) therapy (Im et al., 2016). It was found that intracellular cGAS and STING maintain the stemness of CD8^+^ T cells through upregulation of TCF1 expression and are essential for their functional maintenance (Li et al., 2020). The cGAS–STING pathway-mediated IFNs maintain TCF1 expression in CD8^+^ T cells by suppressing AKT, whereas deletion of cGAS significantly suppresses TCF1. Meanwhile, exogenous IFN-β partially restores TCF1 expression in cGAS-deficient CD8^+^ T cells. Thus, cGAS maintains the stemness of T cells and promotes the differentiation of stem T cells into effector T cells upon stimulation, promoting the antitumor effects of ICIs (Figure 3).

### cGAS regulates apoptosis

Studies have shown that DNA damage-mediated apoptosis induced by physical and chemical factors such as UV irradiation and cisplatin intervention is also dependent on the cGAS–STING pathway (Li et al., 2021a). A low dose of HSV-1 infection activates the cGAS–STING pathway in mouse brain immune cells and induces IFN-β expression, while a high dose of HSV-1 infection activates Caspase-3-dependent apoptotic pathway, causing apoptosis of microglia and other immune cells (Reinert et al., 2021). Furthermore, inhibition of the cGAS/STING/NLRP3 pathway reduces the apoptosis of primary nucleus pulposus cells induced by hydrogen peroxide stimulation and attenuates intervertebral disc degeneration (Tian et al., 2020). In addition, cGAS can promote the apoptosis of tumor cells caused by the cellular mitotic blockade. Because cGAS binds to nucleosomes in a different manner from that when it binds to DNA, cGAS has the affinity for nucleosomes twice as much as that for DNA. During the mitotic arrest of tumor cells induced by anticancer drugs such as paclitaxel, cGAS prefers to bind to nucleosomes, which in turn activates the enzymatic activity to synthesize cGAMP. cGAMP activates the downstream STING–IRF3 signaling pathway, and phosphorylated IRF3 accumulates slowly in cells, relieving the inhibitory effect of Bcl-xL on mitochondrial outer membrane permeability and causing tumor cell apoptosis. This apoptosis-inducing function of cGAS enhances the killing effect of paclitaxel on cervical, lung, and breast cancers in a mouse xenograft tumor model (Zierhut et al., 2019; Figure 3).

### Inhibition of angiogenesis by cGAS

cGAS also plays a unique regulatory role in vascular neogenesis (Yuan et al., 2017). Palmitic acid treatment induces mitochondrial damage, and mtDNA enters the cytoplasm to be recognized by cGAS, which in turn activates the cGAS–STING–IRF3 pathway. The activated IRF3 enters the nucleus and binds to the promoter of the Mst1 gene, inducing Mst1 expression, which in turn leads to Mst1 upregulation, yes-associated protein inactivation, and ultimately the inhibition of angiogenesis. The cGAS–STING pathway-mediated inhibition of angiogenesis may be associated with impaired angiogenesis and wound healing in diabetic patients (Figure 3).

### cGAS inhibits cell proliferation

Cytoplasmic DNA is a universal DAMP. A recent work reported that mtDNA activates cGAS signaling and inhibits YAP-mediated endothelial cell proliferation, thereby promoting inflammatory injury. The bacterial endotoxin LPS activates the perforating protein Gasdermin D to form mitochondrial pores and induces the release of mtDNA into the cytoplasm of endothelial cells. Sensing of mtDNA by cGAS generates a second messenger, cGAMP, which inhibits endothelial cell proliferation by downregulating YAP1 signaling. Therefore, the regenerative capacity of surviving endothelial cells in inflammatory injury is impaired. In an experimental model of inflammatory lung injury, loss of cGAS in mice restores endothelial regeneration. These results suggest that targeting the cGAS–YAP signaling pathway activated by endothelial Gasdermin D may serve as a potential strategy for restoring endothelial function after inflammatory injury (Huang et al., 2020; Figure 3).

### cGAS enhances ICI-mediated antitumor effect

In recent years, blockade of immune checkpoints has achieved remarkable success in cancer therapy. However, most tumor patients do not respond to ICI therapy or even experience hyper progressive disease. Recent work demonstrated that activation of the cGAS–STING signaling pathway significantly promotes the therapeutic effect of ICIs (Wang et al., 2017; de Mingo Pulido et al., 2021). Deletion of *Cgas* or *Sting* results in the failure of anti-PD-L1 therapy in a melanoma transplant model. Further studies found that cGAMP treatment enhances the efficacy of anti-PD-L1 therapy manifesting with improved tumor suppression and survival in mice. Mechanistic studies revealed that cGAMP promotes the maturation of dendritic cells in a cGAS–STING-dependent manner and facilitates the presentation of tumor-associated antigens, thus promoting the killing of tumor cells by tumor-specific antigen-activated CD8^+^ T cells (Wang et al., 2017). A very recent work demonstrated that the blockade of another immune checkpoint, T cell immunoglobulin and mucin-containing molecule 3 (TIM-3), increases the endocytosis of extracellular DNA by dendritic cells and promotes the activation of the cGAS–STING pathway, which in turn enhances the killing effect on tumor cells. In contrast, the effect of anti-TIM-3 treatment alone and its combination with paclitaxel is suppressed after inhibition of cGAS and STING (de Mingo Pulido et al., 2021). These studies suggest that cGAS plays an important regulatory role in ICI-mediated tumor therapy and provides a rationale for the design of a novel cancer therapeutic strategy by combining cGAS–STING activator with ICIs. Notably, as a cGAS agonist, Mn^2+^ has been demonstrated to be powerful anti-tumor agents by promoting the proliferation of CD8^+^ T cells and natural killer cells, as well as the maturation and antigen presentation of dendritic cells, in a cGAS-dependent manner. Moreover, Mn^2+^ produces a synergistic effect in combination with anti-PD-1 treatment, radiotherapy, or chemotherapy (Lv et al., 2020; Figure 3).

### cGAS regulates the DNA replication process

DNA replication is tightly regulated and abnormal DNA replication often leads to the development of various diseases. During DNA replication, DNA-unwinding enzymes open the DNA double strand, and subsequently, multiple replication-related proteins and enzymes bind to the DNA double strand-unwinding site to form replication forks. The replication fork moves along the DNA and completes the replication of DNA. It has been shown that cGAS can regulate DNA replication process in cells (Chen et al., 2020). cGAS was found to inhibit the proliferation of normal and tumor cells, and this inhibition was dependent on its nuclear localization and DNA-binding ability, not on its enzymatic activity and downstream STING proteins. Further mechanistic studies revealed that cGAS in the nucleus interacts with replication fork proteins in a DNA binding-dependent manner, thereby reducing the rate of replication fork advancement and maintaining replication fork stability. When cGAS is absent, the rate of replication fork advancement increases significantly and replication fork stability decreases, resulting in cells in a replication-pressed state. Cells in this state have significantly increased the sensitivity to radiotherapy and chemotherapy, which provides a new theoretical basis for sensitizing cancer chemotherapy (Figure 3).

### cGAS regulates DNA damage repair process

In living organisms, DNA is susceptible to damages caused by endogenous or exogenous factors, and DNA repair is one of the important ways to mitigate the harmful reactions caused by DNA damage. There are five main DNA repair pathways—base excision repair, nucleotide excision repair, mismatch repair, HR, and NHEJ. These repair processes are critical for maintaining intracellular genetic stability and genomic stability (Chatterjee and Walker, 2017). Our study demonstrated that nuclear cGAS inhibits the DNA damage repair process and promotes tumorigenesis (Liu et al., 2018). When DNA DSBs occur, the cytosolic cGAS translocates to the nucleus, where it binds to phosphorylated H2AX through its C-terminal structural domain, which in turn recruits cGAS to reach the DNA damage site. At the site of DNA DSBs, cGAS impairs the formation of the PAPR1/Timeless complex, which inhibits HR repair of DNA DSBs and leads to increased genomic instability. In line with this observation, a later study demonstrated that cGAS inhibits HR repair of DNA damage, resulting in increased micronucleus frequency, increased genomic instability, and ultimately cell death (Jiang et al., 2019; Figure 3).

### cGAS is a micronucleophagy receptor

Micronuclei are extra-nuclear chromosomal structures surrounded by the nuclear membrane, which originate from mutations in chromosomes or genomes. At the end of mitosis, missegregated chromosomes lag behind normal chromosomes as they move toward the cell poles and form an isolated micronucleus because they cannot be encapsulated into the nucleus (Guo et al., 2020). Increased frequency of micronuclei has been found to be associated with various serious diseases, such as malignancies (Guo et al., 2021), inflammatory and autoimmune diseases (Kirsch-Volders et al., 2020), diabetes and obesity (Franzke et al., 2020), chronic kidney disease, and cardiovascular disease (Fenech et al., 2020). *In vitro* studies have shown that cGAS can be recruited to the micronucleus and senses DNA released from ruptured micronucleus to trigger innate immune responses (Mackenzie et al., 2017). This recognition may be inhibited by TREX1, a 3′–5′ DNA exonuclease associated with the ER (Mohr et al., 2021). Induction of micronucleus autophagy is one of the important ways to degrade micronuclei (Guo et al., 2021). Our very recent work demonstrated that cGAS is an autophagy receptor for micronuclei and important for the homeostatic regulation of micronuclei (Zhao et al., 2021). The deletion of cGAS in cells can increase the abundance of micronuclei, and conversely overexpression of cGAS can significantly decrease the number of micronuclei formed spontaneously or induced by genotoxic agents. *In vivo* studies showed that cGAS deletion significantly increased the frequency of nocodazole-induced micronuclei in reticulocytes. Mechanistic studies revealed that cGAS can directly interact with LC3 through its own LC3-interacting region located at the position 355–360, which in turn transports micronuclei to lysosomes for degradation. cGAS-mediated micronuclus autophagic degradation can significantly reduce the intracellular concentration of cGAMP, thereby avoiding excessive activation of innate immunity (Figure 3).

### cGAS regulates metabolism

Recent studies have shown that the cGAS–STING signaling pathway plays a key role in regulating cell metabolism. Obesity can lead to insulin resistance and metabolic diseases such as cardiovascular disease and type 2 diabetes (Wu and Ballantyne, 2020). The activation of the cGAS–STING pathway by DNA may lead to obesity and further promote metabolic disorders in the body (Bai and Liu, 2019). Obesity leads to the release of mtDNA to the cytoplasm, which leads to an inflammatory response by activating the cGAS–STING signaling pathway. Meanwhile, in the context of high-fat diet, fat can specifically overexpress DsbA-L or inhibit the cGAS–STING signaling pathway to promote fat thermogenesis and protect mice from high fat-induced obesity (Bai et al., 2017). IRF3 was found highly expressed in fat cells of obese mice and humans, leading to insulin resistance in fat cells (Kumari et al., 2016). A very recent work found that bacterial DNA can be enriched in β cells of obese individuals. Extracellular vesicles containing intestinal microbial DNA could easily pass through the intestinal barrier of obese individuals and deliver microbial DNA to β cells, which in turn activated the cGAS–STING signaling pathway leading to elevated inflammation and impaired insulin secretion (Gao et al., 2022). In addition, abnormal activation of cGAS–STING induced non-alcoholic steatohepatitis (NASH) in the context of mitochondrial dysfunction. IRF3 was demonstrated to be critical for driving hepatocyte death in alcoholic liver disease, which also induced a strong secondary inflammatory response that affected adjacent cells by regulating NF-κB signaling, inflammatory cytokines, and apoptosis signaling, ultimately leading to liver failure (Qiao et al., 2018). Alcohol-fed mice were reported to have higher levels of cGAS–IRF3 pathway activation in liver cells. Mice that were simultaneously genetically engineered to have lower levels of cGAS and IRF3 expression were less susceptible to alcoholic liver disease (Luther et al., 2020). Taken together, these results suggest that abnormal activation of cGAS–STING signaling may lead to obesity-induced insulin resistance and metabolic disorders, as well as the occurrence of NASH (Figure 3).

## Concluding remarks

Although many advances in the research of cGAS have been made, the functional roles of cGAS still warrant further investigations. The cellular distribution of cGAS may provide hints for its biological functions. PIPs-mediated cell membrane localization of cGAS has been reported to be critical to avoid the activation of cGAS by its own trace DNA (Barnett et al., 2019). However, it remains to address whether membrane cGAS exerts other functions beyond sensing DNA. Our previous work demonstrated that cGAS facilitates the sensing of extracellular cyclic dinucleotides (Liu et al., 2019a), which may be partly explained by the membrane localization of cGAS. The identification of novel membrane proteins associated with cGAS by mass spectrometry will help to further understand the function of membrane cGAS. Much more attention has been paid to the function of nuclear cGAS (Bai and Liu, 2022). Nuclear cGAS has been reported to regulate DNA damage repair and DNA replication fork formation as well as chromosome fusion in STING-independent manner. However, the chromosome-binding property of cGAS makes it appealing to interrogate the functional roles of cGAS in the regulation of chromosomal accessibility and gene transcription, which is partly hindered by the availability of an ideal antibody for the chromatin immunoprecipitation sequencing assay of the cGAS–binding region in the genome. Moreover, though various chemical modulators have been developed for targeting cGAS to modulate its DNA-sensing activity, little progress has been made in targeting cGAS to interfere with functions other than DNA sensing. The increasing understanding of the precise regulation of the cGAS function will help to address these questions.
